# Noninvasive Analysis of Peptidoglycan from Living Animals

**DOI:** 10.1021/acs.bioconjchem.4c00007

**Published:** 2024-04-09

**Authors:** Karl L. Ocius, Sree H. Kolli, Saadman S. Ahmad, Jules M. Dressler, Mahendra D. Chordia, Brandon L. Jutras, Melanie R. Rutkowski, Marcos M. Pires

**Affiliations:** † Department of Chemistry, University of Virginia, Charlottesville, Virginia 22904, United States; ‡ Department of Microbiology, Immunology, and Cancer Biology, University of Virginia, Charlottesville, Virginia 22904, United States; § Department of Biochemistry, Virginia Tech, Blacksburg, Virginia 24061, United States; ∥ Fralin Life Sciences Institute, Virginia Tech, Blacksburg, Virginia 24061, United States; ⊥ Center for Emerging, Zoonotic and Arthropod-borne Pathogens, Virginia Tech, Blacksburg, Virginia 24061, United States

## Abstract

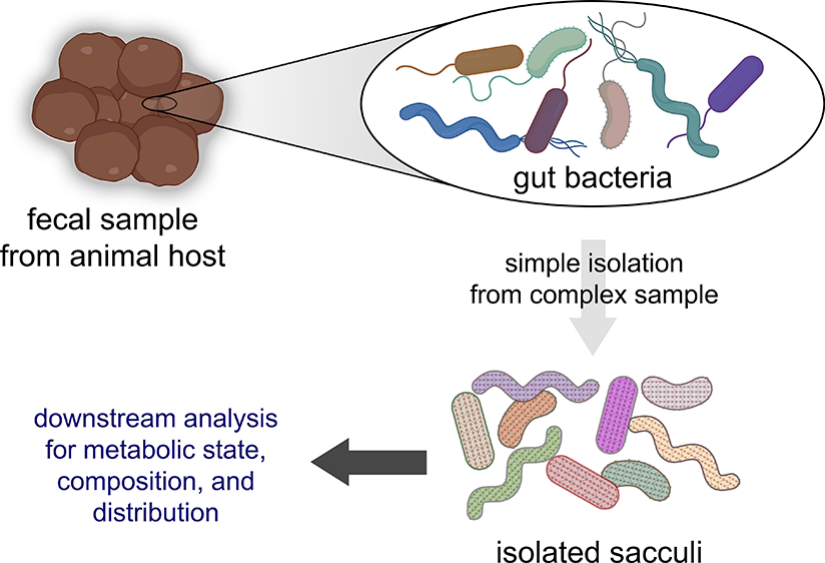

The role of the intestinal microbiota in host health is increasingly revealed in its contributions to disease states. The host-microbiome interaction is multifactorial and dynamic. One of the factors that has recently been strongly associated with host physiological responses is peptidoglycan from bacterial cell walls. Peptidoglycan from gut commensal bacteria activates peptidoglycan sensors in human cells, including the nucleotide-binding oligomerization domain-containing protein 2. When present in the gastrointestinal tract, both the polymeric form (sacculi) and depolymerized fragments can modulate host physiology, including checkpoint anticancer therapy efficacy, body temperature and appetite, and postnatal growth. To utilize this growing area of biology toward therapeutic prescriptions, it will be critical to directly analyze a key feature of the host-microbiome interaction from living hosts in a reproducible and noninvasive way. Here we show that metabolically labeled peptidoglycan/sacculi can be readily isolated from fecal samples collected from both mice and humans. Analysis of fecal samples provided a noninvasive route to probe the gut commensal community including the metabolic synchronicity with the host circadian clock. Together, these results pave the way for noninvasive diagnostic tools to interrogate the causal nature of peptidoglycan in host health and disease.

## Introduction

**Figure 1:**
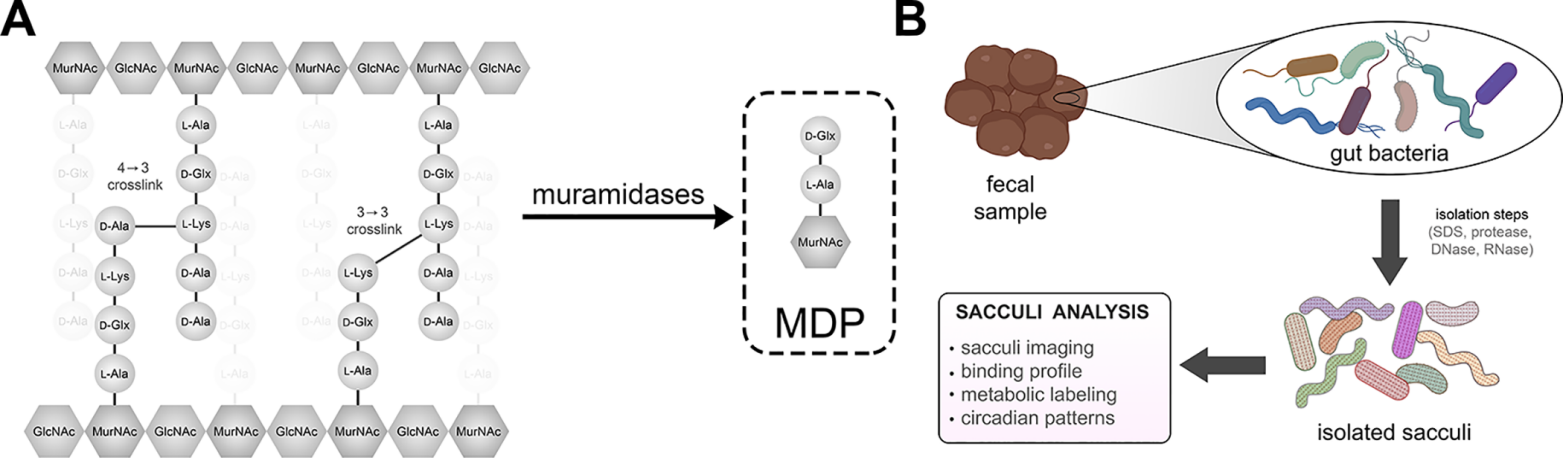
(A) Cartoon representation of the interaction between gut microbiota and intestinal lining. This interaction can be driven by the two-way distribution of signaling molecules from and to the host. There are a few molecules that have been identified that are released by gut bacteria, which are known to modulate host physiology. (B) Workflow for the analysis of sacculi from fecal samples. Samples are subjected to a series of treatments that ultimately result in the isolation of sacculi that can be analyzed with downstream assays.

The human gastrointestinal (GI) tract is populated by a community of microorganisms that are purported to be involved in a range of biological functions, such as synthesizing vitamins, training the immune system, and protecting the host against pathogens.^1−5^ Disruptions to the human microbiota have been linked to a range of health dysfunctions, including digestive disorders, autoimmune diseases, and obesity. This complex relationship between the microbiota and the host is mediated by multiple factors including the exchange of biologically active molecules. Bacterial products interact with the host immune cells in the gut and influence the development and function of the immune system, which can impact host susceptibility to infections, response to pathogenicity (including cancer), and the development of autoimmune diseases. Recent evidence has revealed that bacterial cell walls are, in fact, biologically active agents to their host organisms (Figure 1A).^6−8^

Bacterial peptidoglycan is a major component of bacterial cell walls, playing a key role for the defense of bacterial cells.^9,10^ The peptidoglycan scaffold surrounds the entire cell, and this single molecule is known as the bacterial sacculus. Peptidoglycan is composed of unique building blocks that are not present in humans, including a polymeric glycan backbone built from disaccharides of *N*-acetylglucosamine (GlcNAc) and *N*-acetylmuramic acid (MurNAc). A short peptide (the stem peptide) is connected to each MurNAc unit and is cross-linked across neighboring chains forming a mesh-like structure. The stem peptide can vary in composition but is typically 5 amino acids in length, with the sequence l-Ala-*iso*-d-Glu-l-Lys (or *meso*-diaminopimelic acid [*m*-DAP])-d-Ala-d-Ala.^11^ Given the unique chemical composition of peptidoglycan, it represents an ideal biomarker to indicate the presence of bacteria in any system.^10,12^

Organisms have evolved diverse strategies to sense the presence of peptidoglycan as a mode of self-defense and microbiome maintenance.^13−15^ These include peptidoglycan recognition proteins (PGRPs)^16^ and lysin motif (LysM) domains that recognize polymeric peptidoglycan,^17,18^ and nucleotide-binding and oligomerization domain proteins (NOD1/NOD2) that recognize peptidoglycan fragments.^19−22^ For some receptors, the primary function could be to alert the immune system to the presence of a dangerous bacterial pathogen and trigger an inflammatory host response,^9^ including for fragments of peptidoglycan.^23,24^ More recently, a plethora of studies have provided evidence that fragments of peptidoglycan can lead to positive outcomes for the host via NOD2 signaling by agonists, such as peptidoglycan fragments (e.g., muramyl dipeptide, MDP^25−27^).^6,8,28−30^ Peptidoglycan fragments were recently shown to significantly improve responses to checkpoint inhibitors in a cancer model in mice,^30^ modulate body temperature and appetite of mice,^31^ and alleviate Crohn’s disease phenotypes.^8,32^ Critically the health effects were not limited to peptidoglycan fragments; the administration of purified bacterial sacculi from *Lactobacillus plantarum* (*L. plantarum*) led to significant growth improvement in undernourished mice.^6^ Additionally, the impact of sacculi on host biology may not be confined to interactions in the GI tract. Radiolabeled sacculi that were orally administered to mice translocated to the circulatory system and led to subsequent systemic dissemination.^33^

Despite the growing appreciation that gut bacterial sacculi (and their peptidoglycan fragments) are bona fide interspecies signaling molecules,^34,35^ the direct characterization of polymeric sacculi from living organisms has not been extensively evaluated. The Boneca laboratory used radiolabeled sacculi that were labeled in vitro to define the translocation of peptidoglycan into host tissues.^33^ Given the vast diversity of the gut microbiota, including a large fraction that has yet to be cultured in vitro, the isolation of sacculi from gut bacterial communities may represent our most reliable access point to polymeric precursors to these peptidoglycan-host signaling molecules. To address the role of sacculi in gut microbiota health, a necessary first step is to directly interrogate sacculi from a living host via a noninvasive and user-friendly approach.

Here, we directly isolated sacculi from stool samples to gain insight into the status of the gut microbiome community following downstream analysis including structural analysis, analysis with binding probes, and metabolic tagging (Figure 1B). To date, stool samples have provided the most accessible and least disruptive method to examine the gut microbiome community from a living host. To this end, a large majority of the important studies linking the gut microbiota with various host (human and mice) health impacts have relied on fecal analysis.^36−40^ Our results showed the feasibility of readily isolating sacculi from fecal samples collected from mice and humans; results that create the opportunity to couple sacculi analysis to gut bacterial status. Furthermore, we site selectively tagged peptidoglycan of gut bacteria in live host and recovered them in the fecal samples. We project that the metabolic tags can be leveraged to monitor cell wall biosynthesis/remodeling dynamics in the context of changing external conditions (including light-dark cycles).

## Results

The GI tract has the largest concentration of commensal microorganisms in mammalian hosts such as mice and humans. While analysis by whole genome sequencing from fecal samples has become routine and it affords species level identification of the microbial community, it has been a challenge to turn this wealth of data toward functional assays. In contrast, to the best of our knowledge, there have not been attempts to isolate and leverage the sacculi of bacteria from stool samples. A unique physicochemical property of sacculi is their resistance to a range of degrative enzymes (proteases, DNases, and RNases) and detergents; this feature enables the robust and highly reproducible isolation of pure peptidoglycan from cultured cells. We reasoned that this workflow could also be translated to selectively isolate sacculi from the highly complex stool sample matrix. In doing so, we hypothesized that it would be possible to leverage this important biopolymer as a biomarker of the host-microbiota peptidoglycan-signaling axis.

**Figure 2:**
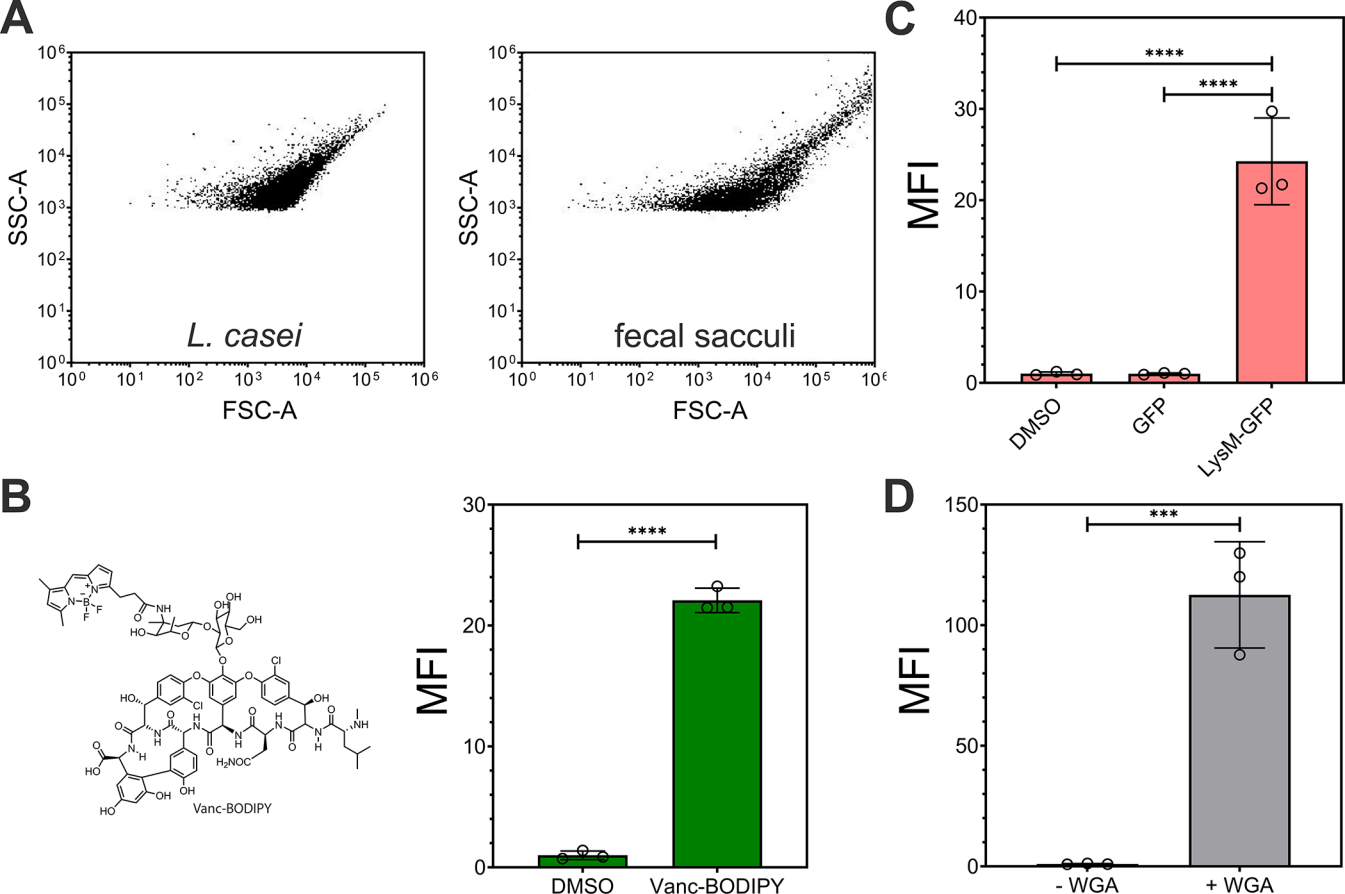
(A) Forward and side scatter plots of sacculi isolated from *L. casei* cultured in vitro and fecal sacculi from mice. (B) Flow cytometry analysis of sacculi isolated from fecal samples of mice in the presence of DMSO or Vanc-BODIPY (2 μg/mL) for 60 min then washed with PBS. (C) Flow cytometry analysis of sacculi isolated from fecal samples of mice in the presence of Lysm-GFP, GFP, or no protein (1 μΜ) for 60 min then washed with PBS. (D) Flow cytometry analysis of sacculi isolated from fecal samples of mice in the presence of DMSO or fluorescein WGA (1 μM) for 60 min then washed with PBS. Mean fluorescence intensity (MFI) is the ratio of fluorescence levels above the control (DMSO) treatment from 10000 events. *P*-values were determined by a two-tailed *t*-test (* denotes a *p*-value <0.05, ** < 0.01, and *** <0.001, ns = not significant).

To start, fecal samples from specific pathogen-free (SPF) adult mice were collected and subjected to standard sacculi isolation procedures that are performed in vitro. SPF mice have a normal gut microbiome that are free of some specific pathogens. The sample is initially boiled in sodium dodecyl sulfate (SDS), followed by treatment with trypsin, DNase, and RNase. We previously showed that sacculi from cells cultured in vitro can be readily analyzed by flow cytometry given that their size mirrors that of live bacterial cells.^41^ As expected, the scatter profile of the fecal sacculi sample was similar to that of sacculi from *Lactobacillus casei* (*L. casei*) (Figure 2A). *L. casei* is part of the natural flora of humans and can be used as a model organism.^42^ The sacculi sample was then subjected to a number of treatments with agents that have affinity toward the stem peptide or the saccharide backbone on bacterial sacculi to confirm the composition of the isolated biopolymer; this process resulted in approximately 32 mg of sacculi/g of stool sample (dry weight).

Vancomycin is a glycopeptide that selectively binds to the terminal d-Ala-d-Ala motif on the stem peptide of the peptidoglycan. When linked to the fluorescent dye BODIPY, this conjugate labels whole cells and isolated sacculi.^43^ Incubation of the fecal sacculi sample with Vanc-BODIPY led to a large shift in fluorescence levels, an indication that the material contained the d-Ala-d-Ala motif, which is consistent with isolated sacculi (Figure 2B). As expected, a similar profile was also observed using the sacculi from cultured *L. casei* (Figure S1). While vancomycin is only biologically active against live Gram-positive bacteria, the sacculi isolation steps will expose d-Ala-d-Ala on sacculi isolated from both Gram-positive and -negative bacteria. To demonstrate that the fluorescent signals were representative of this binding event, sacculi were also treated with a synthetic analog of the stem peptide, l-Lys-d-Ala-d-Ala. A competition experiment was performed by cotreatment of sacculi with Vanc-BODIPY and l-Lys-d-Ala-d-Ala. Accordingly, lower levels of fluorescence were observed upon the addition of l-Lys-d-Ala-d-Ala in a concentration-dependent manner (Figure S2). These studies provided evidence that the isolated material had binding signatures consistent with the stem peptide of sacculi.

Two additional sacculi binding reagents were tested next to probe the inclusion of the disaccharide backbone within the isolated biopolymers. LysM domains, which are widely found in nature, bind to the GlcNAc saccharide^44^ motif in the backbone of peptidoglycan.^45^ Isolated LysM domains have been shown to bind whole cells and isolated sacculi.^17,46,47^ Treatment of sacculi from fecal samples with LysM fused to GFP led to an increase in fluorescence associated with the sacculi (Figure 2C). Treatment with GFP alone and DMSO resulted in near background levels of fluorescence. Similarly, cells were treated with wheat germ agglutinin (WGA) modified with a fluorescein fluorophore. WGA, also known to bind GlcNAc, was recently shown to bind to sacculi of *Borrelia burgdorferi*.^48^ Treatment of the fecal sacculi with fluorescent WGA resulted in a 120-fold increase in fluorescence, which suggests that the material has GlcNAc (Figure 2D), similar to that of lactobacilli cultured in vitro (Figure S3). The use of excess monomeric GlcNAc resulted in a statistically significant lowering of fluorescence levels, an indicative of the competition of GlcNAc for WGA (Figure S4). Additionally, we tested the binding of the cells to soybean agglutinins (SBA) and found that the signals were greatly diminished as expected given their binding specificity (Figure S5).

**Figure 3:**
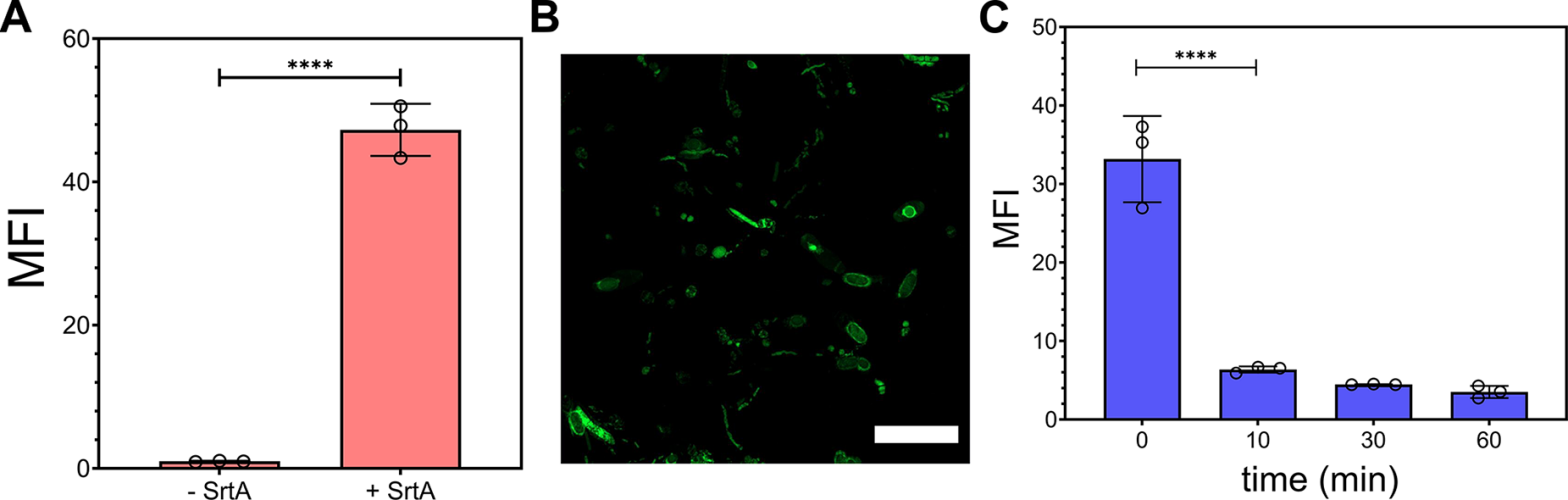
(A) Flow cytometry analysis of sacculi isolated from fecal samples of mice in the presence of 100 μM Fl-LPMTG (substrate) and SrtA (20 μM) or without SrtA for 5 h then washed with 100 mM Tris, 5 mM EDTA, pH 7 and freshly added 8 M urea. (B) Confocal microscopy of sacculi isolated from fecal samples of mice after tagging with Fl-LPMTG and SrtA; scale bar = 10 μm. (C) Flow cytometry analysis of sacculi isolated from fecal samples of mice after tagging with Fl-LPMTG and SrtA in the presence of mutanolysin (25 μg/mL). Samples were monitored across varying time points. Mean fluorescence intensity (MFI) is the ratio of fluorescence levels above the control (-SrtA) treatment from 10,000 events. *P*-values were determined by a two-tailed *t*-test (* denotes a *p*-value <0.05, ** < 0.01, *** <0.001, and **** <0.0001, ns = not significant).

Appreciating that peptidoglycan stem peptides may have glycine residues^49^ and also remaining from the trypsin digestion of proteins that are covalently attached to the peptidoglycan (e.g., sortase^50^ anchored or Braun’s protein^51^), we reasoned that we could enzymatically install probes onto the fecal sacculi using Sortase A from *Staphylococcus aureus*. Sortase A (SrtA) is a transpeptidase that covalently anchors endogenous proteins onto the stem peptide of the peptidoglycan scaffold. More specifically, SrtA recognizes the LPXTG (where X is any amino acid) motif to link the third position amino acid on the stem peptide between T and G on the anchored protein. We^41,52,53^ and others^54^ have previously used synthetic analogs of LPXTG conjugated to a fluorophore on the *N*-terminus to metabolically tag isolated bacterial sacculi from pure culture. Here, we incubated fecal sacculi with Fl-LPMTG (fluorescein-linked LPMTG, Figure 3) in the presence of SrtA and saw a 46-fold increase in fluorescence associated with the sacculi (Figure 3A). In the absence of SrtA, basal levels of fluorescence were observed when fecal sacculi were also coincubated with Fl-LPMTG. Similar fluorescence profiles were also observed with sacculi from *L. casei* (Figure S6). The same SrtA fecal sacculi sample was analyzed by confocal microscopy, and the sacculi of the fecal sample matched well with prior profile of whole cells extracted from fecal samples of mice (Figure 3B).^55^ Together, we showed that we can use bacteria-specific enzymes to covalently tag the sacculi of gut microbiota harvested from the fecal sample.

Given the polymeric saccharide backbone of peptidoglycan, it may be susceptible to digestion by muramidases such as lysozyme and mutanolysin. *O*-acetylation of muramic acid can result in lysozyme resistance.^56^ Considering the evidence that there is better structural coverage of fecal samples with mutanolysin than lysozyme^57^ in the cell disruption step and that there is evidence that some gut bacteria include *O*-acetylation,^58^ we chose to test muramidase activity with mutanolysin. Sacculi that had been prelabeled with Fl-LPMTG were incubated with mutanolysin, and fluorescence levels were periodically measured. Our data showed that there is a rapid decrease in fluorescence within 10 min, a clear indication that the isolated material is peptidoglycan in nature (Figure 3C). Combined, this broad range of techniques provided foundational evidence that the isolated biopolymer from the fecal samples of mice was primarily sacculi.

**Figure 4:**
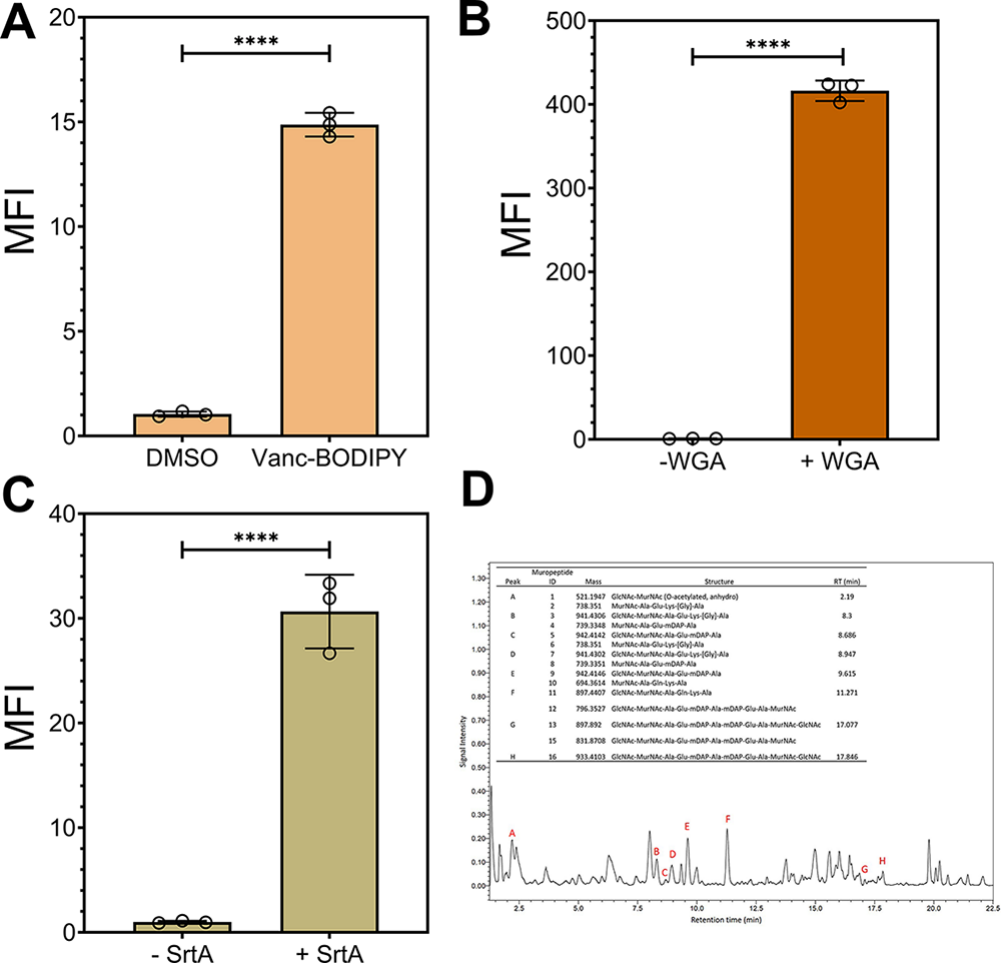
(A) Flow cytometry analysis of sacculi isolated from fecal samples of humans in the presence of DMSO or Vanc-BODIPY (2 μg/mL) for 60 min then washed with PBS. (B) Flow cytometry analysis of sacculi isolated from fecal samples of humans in the presence or absence of WGA (1 μM) for 60 min then washed with PBS. (C) Flow cytometry analysis of sacculi isolated from fecal samples of mice in the presence of 100 μM Fl-LPMTG (substrate) and SrtA (20 μM) or without SrtA for 5 h then washed with 100 mM Tris, 5 mM EDTA, pH 7, and freshly added 8 M urea. (D) LC-MS chromatogram of processed human fecal samples (black). Muropeptide-containing peaks are labeled (red), and corresponding muropeptide identities (inset table) are shown. MS2 spectra for each peak were scanned for (204.085 *m*/*z*) and a GlcNAc ring cleavage product (138.053 *m*/*z*), and ion masses were compared to known muropeptide masses to assign identity. Data shown represent the results attained from two technical replicates.

Having established sacculi can be readily isolated from the fecal pellets of mice, we sought to demonstrate that a similar workflow of steps should be readily adoptable to human stool samples. In human research, stool samples are conveniently deployed as the primary biospecimen to evaluate the composition and functionality of the human gut microbiota. This is primarily due to the abundance of biomass and the practicality of collection, and it has been shown to serve as a valuable proxy for investigating the luminal gut microbiome.^1^ We obtained fecal samples (deidentified) from human donors and matched the steps we had established from mouse fecal samples to isolate the bacterial sacculi. Satisfyingly, large increases in fluorescence levels were observed through the treatment of Vanc-BODIPY, WGA, and SrtA (Figure 4A–C). These results suggest that the sacculi from fecal samples can be conveniently isolated in the course of approximately 8 h, which provides a potential handle to interrogate the health of a human patient in the event of a pathology that is linked to gut microbiota disturbance. As expected, there is inherently heterogeneity in labeling levels in a complex mixture of sacculi harvested from the microbiota of a human host. These differences are expected to be observed in the size of the sacculi, the thickness of the matrix, and the chemical diversity (amino acids, disaccharide backbone, crossbridge levels, amidation levels, etc.); together, these features can impact the interactions with Vanc-BODIPY and WGA and processing with SrtA. To this end, we see that there are populations of cells that are labeled relatively weakly and populations that are labeled to much greater extents (Figure S7). Finally, the isolated sacculi were digested with muramidases, and the soluble fragments were analyzed by liquid-chromatography mass spectrometry (Figure 4D). Our results showed that a number of the chromatographic peaks had mass (*m*/*z*) signatures consistent with muropeptides that included some with a lysine or *m*-DAP in the third position. The remaining peptides could potentially be fragments that were covalently anchored to the sacculi by native transpeptidases. These results further confirmed that the isolated insoluble material was enriched in muropeptides.

**Figure 5:**
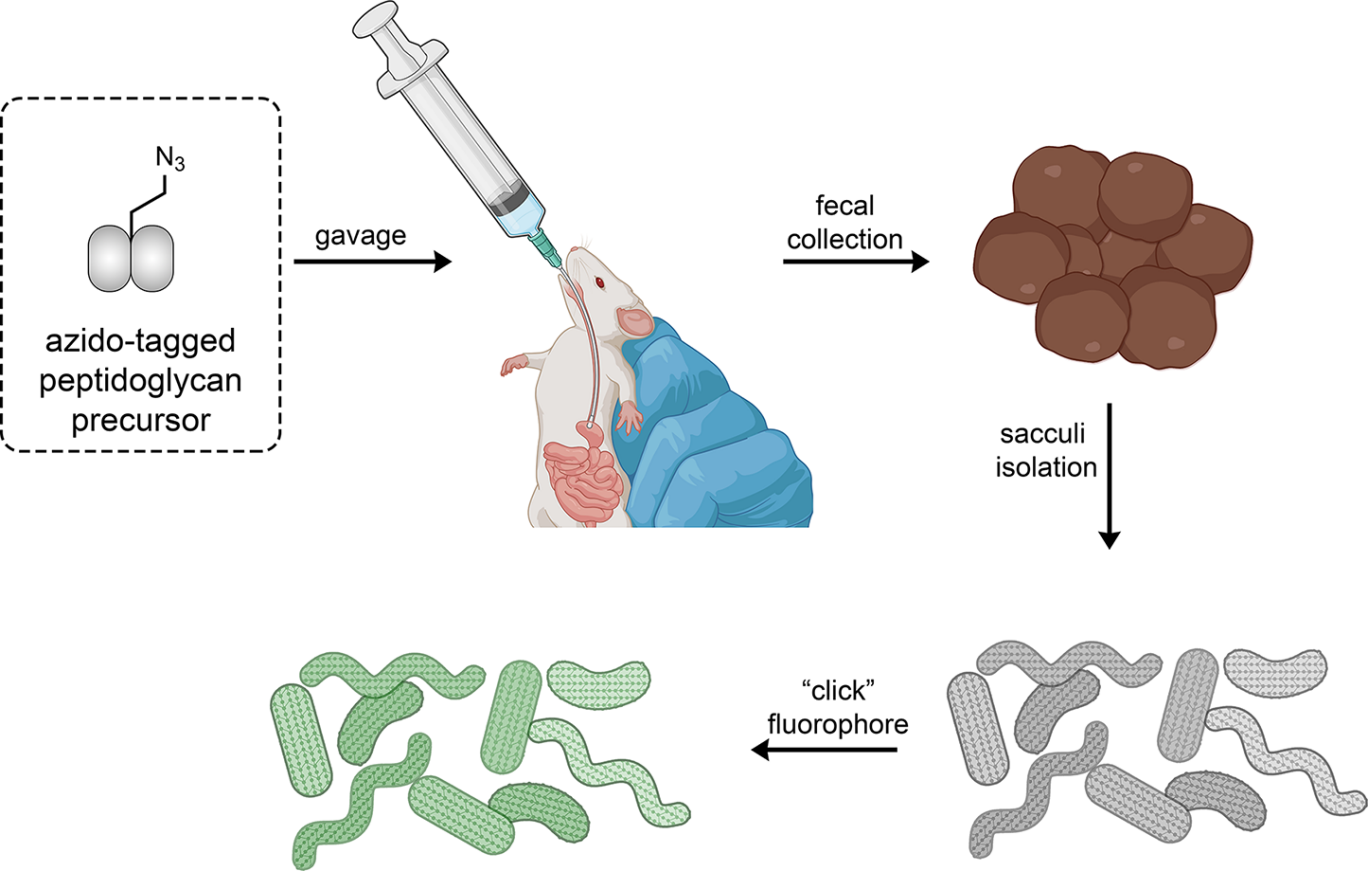
Workflow of the oral administration of a synthetic peptidoglycan analog bearing an azido tag. Following the incorporation of the tag into the cell wall of live bacteria, the fecal samples are collected, and the sacculi are isolated. Finally, a click reaction is performed on the isolated sacculi to install the fluorophore. Sacculi that have been metabolically labeled can subsequently analyzed with various techniques.

We then set out to metabolically label peptidoglycan of gut bacteria in live mice and isolate them directly from fecal samples (Figure 5). The goal was to show that the installation of peptidoglycan-specific probes could be detected by a noninvasive method to monitor the metabolism of gut bacteria. Metabolic labeling of the peptidoglycan of cultured bacteria has been heavily explored for studying cell wall dynamics.^59−69^ The simple incubation of synthetic analogs of peptidoglycan with bacterial cells results in their incorporation by promiscuous cell wall enzymes; analogs can be modified with fluorophores or click handles, thus providing routes to illuminating cell wall biosynthesis. In 2017, Hudak et al. (and since then others^70−72^) conjugated a fluorophore to a D-amino acid metabolic tag and found that this probe labeled bacteria in the gut of mice. At the same time, our laboratory showed in vivo metabolic labeling of peptidoglycan from *Staphylococcus aureus* harbored in *Caenorhabditis elegans*(73) followed by a recent demonstration in mice.^74^

**Figure 6:**
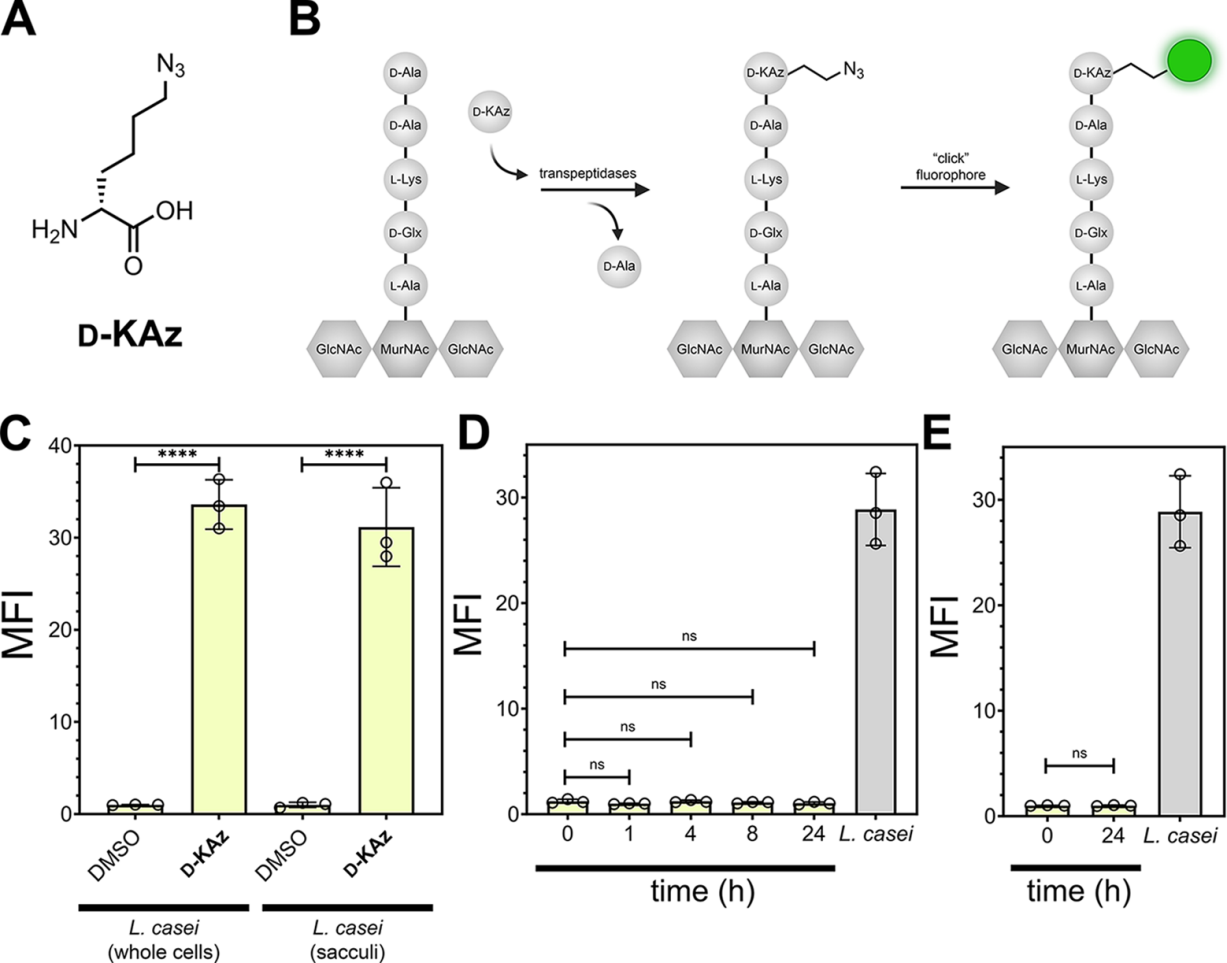
(A) Chemical structure of **D-KAz**. (B) Schematic cartoon representation of the swapping of the terminal d-alanine for the modified D-amino acid in the media. After installation of the azido tag, a click reaction results in a covalent modification with the fluorophore. (C) Flow cytometry analysis of whole cells or sacculi of *L. casei*. *L. casei* cells were treated overnight with 1 mM of **D-KAz** or DMSO then labeled as whole cells by treating with 30 μM of alkyne-fluorescein. Alternatively, cells were subjected to sacculi isolation steps before performing the click reaction. (D) Mice were orally dosed with 5 mM of **D-KAz** 2X 1 h apart in a daylight cycle. Following the second dosing, the animals were moved to a new cage. Fecal samples were collected at the designated times and subjected to sacculi isolation and a click reaction to install the fluorophore. The levels were compared to sacculi of *L. casei* labeled in vitro with **D-KAz**. (E) The cecum contents of the mice from (D) at time 24 h were harvested, bacterial cells were retrieved, and the sacculi were isolated before performing a click reaction with a fluorophore. The levels were compared to sacculi of *L. casei* labeled in vitro with **D-KAz**. Mean fluorescence intensity (MFI) is the ratio of fluorescence levels above the control (DMSO) treatment from 10000 events. *P*-values were determined by a two-tailed *t*-test (* denotes a *p*-value <0.05, ** < 0.01, *** <0.001, and **** <0.0001, ns = not significant).

To initially benchmark the metabolic labeling conditions, *L. casei* was cultured with DMSO or in the presence of **D-KAz** (Figure 6A). **D-KAz** bears an azido tag and it is expected to be metabolically incorporated throughout the peptidoglycan scaffold. Single D-amino acid tags become inserted into the stem peptide of the peptidoglycan by endogenous bacterial transpeptidases, which are responsible for peptidoglycan cross-linking (Figure 6B).^75−77^ The installation of the azido group can be revealed with a copper-catalyzed click reaction to an alkyne-modified fluorescein.^78^*L. casei* cultured in the presence of **D-KAz** led to a 31-fold increase in fluorescence levels relative to untreated cells when cells were analyzed as intact structures (Figure 6C). As an alternative method to show the installation of the azido groups on the peptidoglycan, sacculi were first extracted from *L. casei* grown with DMSO or **D-KAz**. The isolated sacculi were then reacted with the fluorophore, and a similar level of fluorescence increase was observed. These results indicate that the azido groups found in the whole cells are likely exclusively within the peptidoglycan. More importantly, these findings illustrate the chemical stability of the azido tag to the steps associated with sacculi isolation.

With the metabolic conditions benchmarked, we orally administered mice with **D-KAz** to analyze labeled sacculi from stool samples. Mice were dosed two times (1 h apart) with **D-KAz** in PBS, and subsequently, fecal pellets were collected at several time points following the second oral dosing before being subjected to the sacculi isolation steps (Figure 6D). No statistically significant differences in sacculi fluorescence levels were found up to 24 h after dosing of the peptidoglycan tag. Two likely scenarios may have been responsible for these results: the level of metabolic labeling was insufficient under the conditions that were tested and resulted in low levels of azide-tagging, or there had not been enough time for the tagged cells to passage through the gut to the stool sample. To determine if labeled cells had not yet moved to the stool samples, the cecum contents were harvested. Cells found in this region of the GI tract also had sacculi with fluorescence levels near background, suggesting that poor labeling incorporation or retention may have been the reason for lack of fluorescence signals in the sacculi samples (Figure 6D).

**Figure 7:**
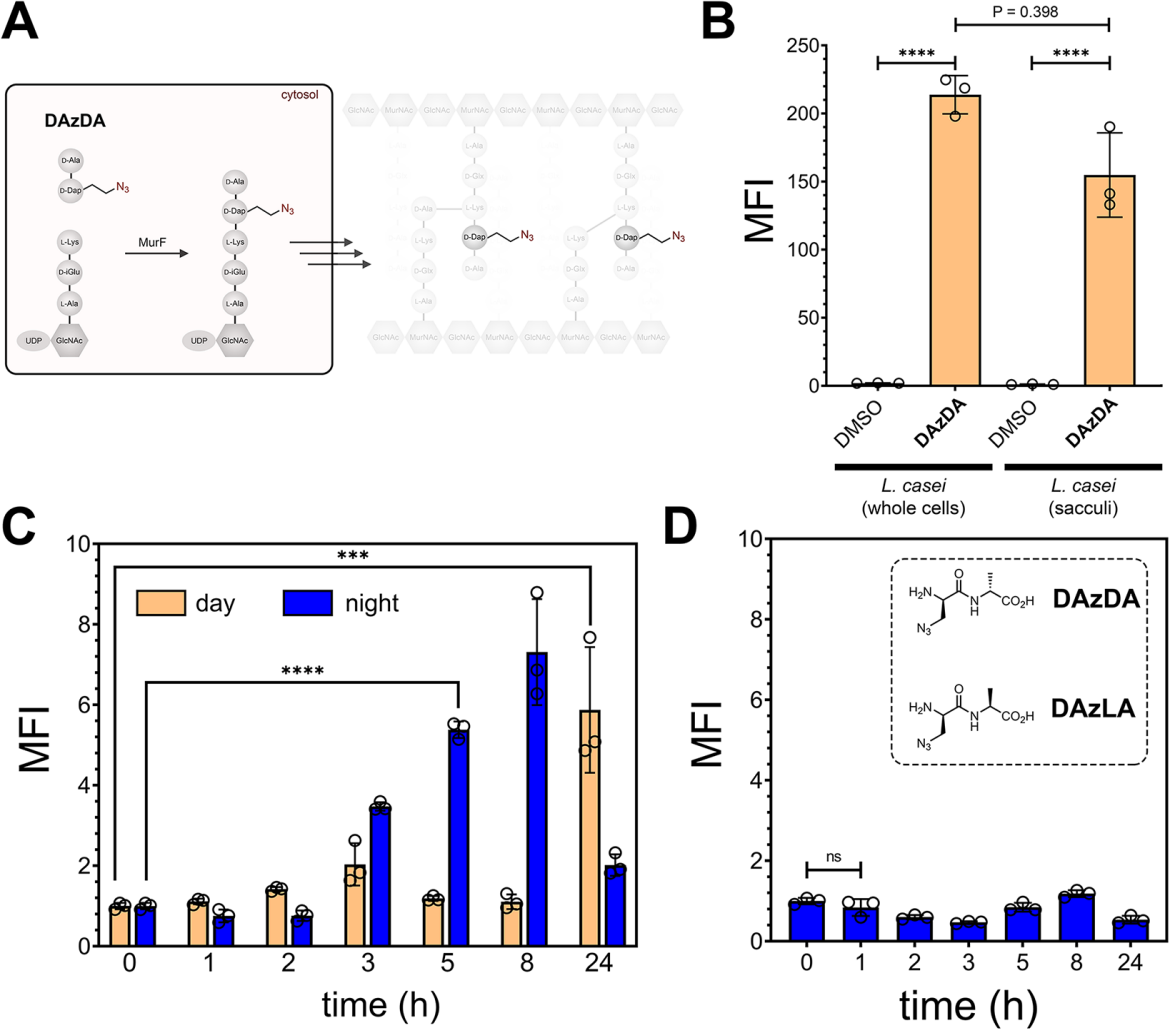
(A) Schematic cartoon showing how synthetic analogs of d-Ala-d-Ala enter the biosynthetic pathway at the MurF ligation step. (B) Flow cytometry analysis of whole cells or sacculi of *L. casei*. *L. casei* cells were treated overnight with 1 mM of **DAzDA** or DMSO and then labeled as whole cells by treating with 30 μM of alkyne-fluorescein. Alternatively, cells were subjected to sacculi isolation steps before performing the click reaction. (C) SPF mice were orally dosed with 5 mM of **DAzDA** 2X 1 h apart. Following the second dosing, the animals were moved to a new clean cage. Fecal samples were collected at the designated times and subjected to sacculi isolation and a click reaction to install the fluorophore. The process was performed during daylight and during the night cycles. (D) SPF mice were orally dosed with 5 mM of **DAzLA** 2X 1 h apart. Following the second dosing, the animals were moved to a new clean cage. Fecal samples were collected at the designated times and subjected to sacculi isolation and a click reaction to install the fluorophore. The process was performed during the night cycle. Mean fluorescence intensity (MFI) is the ratio of fluorescence levels above the control (DMSO) treatment from 10,000 events. *P*-values were determined by a two-tailed *t*-test (* denotes a *p*-value <0.05, ** < 0.01, ***<0.001, and **** <0.0001, ns = not significant).

To increase the overall bacterial cell wall labeling levels, an alternative metabolic tagging strategy was evaluated. Instead of single amino acids, we tested dipeptides that are mimics of the cytosolic peptidoglycan precursor, d-Ala-d-Ala. We^79,80^ and others^64,81^ had previously demonstrated that synthetic d-Ala-d-Ala analogs displaying click chemistry handles (e.g., azide or alkyne) can be installed into peptidoglycan precursors in the intracellular space of bacteria (Figure 7A). Most relevant to the proposed change in labeling strategy, we had previously found that labeling levels were generally higher with dipeptide metabolic tags relative to single amino acids.^79,80^ We synthesized **DAzDA** that displays an azido tag on the alanine side chain of the *N*-terminal amino acid (Figure 7A) and tested its incorporation efficiency in vitro using *L. casei*. Treatment of cells with **DAzDA** led to a 210-fold increase in fluorescence levels compared to untreated whole cells (Figure 7B), which is considerably higher than **D-KAz** labeling levels. As with the single amino acid probe, the azido group was evidently stable through the sacculi isolation steps, and the sacculi fluorescence levels from **DAzDA** labeling were similarly higher. The slightly higher level of fluorescence for the whole cell analysis is likely due to the changing environment of the fluorophore in the two environmental contexts or could be through a minor loss of peptidoglycan through the isolation procedure. Importantly, cellular labeling with **DAzDA** was observed by 30 min after incubation, thus establishing that short incubation times can be sufficient for peptidoglycan incorporation (Figure S8). Finally, confocal microscopy analysis showed that the labeling pattern observed with **DAzDA** was consistent with the expected sacculi structure (Figure S9).

Mice were next dosed with **DAzDA** to probe for improved metabolic tagging of sacculi in live mice. As before, mice were dosed with the metabolic tag twice (1 h apart), and fecal samples were collected following the second administration. In the first round, mice were dosed during the daylight cycle starting early in the morning. Interestingly, the only large difference was between time 0 and time 24 h, without significant change in any of the intervening time points (Figure 7C). We wondered whether the circadian rhythm of the host could be impacting the incorporation of the metabolic tag. After all, the incorporation of the metabolic tag is tightly linked with the overall metabolic processing of the bacterial cell wall. Moreover, it had been shown that the gut microbiome exhibits diurnal variations in its composition.^82,83^ Certain microbial species and their metabolic activities display rhythmic patterns that are influenced by the circadian clock of the host. For example, the abundance and diversity of specific bacteria in the gut can vary throughout the day. To test the possibility that metabolic labeling of the sacculi levels could be impacted by circadian clocks, **DAzDA** was dosed at night and fecal samples were collected following the last administration as the day experiment. A distinctly different pattern of sacculi metabolic labeling emerged (Figure 7C). A steady increase in sacculi fluorescence levels was observed throughout the night and 5 h following administration, there was a marked increase in sacculi relative to the initial measurements. By 24 h, the fluorescence levels were approaching those of initial measurements. Confocal analysis of samples isolated from in situ labeled bacteria yielded structures consistent with sacculi (Figure S10). As seen with the human samples, there was a marked distribution of labeling levels in the inherently complex microbiota sample from mice (Figure S11).

To test the specificity of the metabolic labeling, a new dipeptide was synthesized that was a diastereomeric analog that contained an L-amino acid on the *C*-terminus (Figure 7C). We^79,80^ previously showed that the stereocenter of d-Ala-d-Ala analogs is crucial for recognition by the biosynthetic machinery. Satisfyingly, mice dosed with **DAzLA** resulted in sacculi with near background fluorescence levels throughout the time sampling time window. These results are consistent with a bacterial-specific incorporation of **DAzLA** in live bacteria cells in the mice. We believe that metabolic labeling of gut microbiota can be a tool to study the metabolic kinetics of gut bacteria in a noninvasive and -disruptive method that can be repeatedly probed over time using stool samples.

## Conclusions

In conclusion, we described the noninvasive analysis and metabolic labeling of bacterial peptidoglycan in the gut microbiota. Fecal samples from mice were subjected to sacculi isolation procedures, and various techniques were employed to examine and label the peptidoglycan. The experiments revealed that the isolated sacculi displayed similar characteristics to those of known bacterial sacculi, confirming their identity. Multiple binding reagents, including vancomycin, LysM domains, and WGA, were used to detect specific components of the peptidoglycan structure and confirm successful sacculi isolation. Additionally, we utilized Sortase A to enzymatically install probes onto the fecal sacculi, and fluorescence assays demonstrated successful incorporation. While harvesting the bulk sacculi from the fecal samples can provide a direct, rapid, and reliable route to an important biomolecule that has implications to host-microbiome interactions, there are also some limitations. The heterogeneity (and also sometimes the similarities) across strains of bacteria that reside within the gut will make it difficult to establish specific bacterial species/populations.

Metabolic labeling of peptidoglycan in live mice was also demonstrated using synthetic peptidoglycan analogs. The results showed specific tagging of sacculi, indicating successful incorporation of the analogs into the peptidoglycan scaffold. Interestingly, the time of dosing influenced the metabolic labeling levels, suggesting a potential impact of the host’s circadian rhythm on bacterial cell wall biosynthesis. Additionally, we demonstrated the feasibility of noninvasive and repeated probing of gut bacterial metabolism using metabolic labeling techniques, providing a potential tool for studying the dynamics of gut microbiota over time. We propose that directly isolating sacculi from stool samples can offer a novel and biologically relevant assessment route to the interaction between gut microbiota and the host.

## Supporting Information Available

In vitro labeling of bacterial cells with peptidoglycan binding reagents, confocal microscopy images of bacterial cells, flow cytometry scatter plots, and materials/methods (PDF)
